# Enhancing the Electrochemical Properties of LaCoO_3_ by Sr-Doping, rGO-Compounding with Rational Design for Energy Storage Device

**DOI:** 10.1186/s11671-020-03411-z

**Published:** 2020-09-24

**Authors:** Bin Zhang, Chuanfu Yu, Zijiong Li

**Affiliations:** 1 School of Physics and Microelectronics, Zhengzhou University, Zhengzhou, 450001 Henan China; 2 Henan Aerospace Hydraulic & Pneumatic Technology Co., Ltd., Zhengzhou, 450011 China; 3 School of Physics & Electronic Engineering, Zhengzhou University of Light Industry, Zhengzhou, 450002 China

**Keywords:** Perovskite oxides, LaCoO_3_-based nanocomposites, Rational design, Electrode materials, Energy storage devices

## Abstract

Perovskite oxides, as a kind of functional materials, have been widely studied in recent years due to its unique physical, chemical, and electrical properties. Here, we successfully prepared perovskite-type LaCoO_3_ (LCOs) nanomaterials via an improved sol-gel method followed by calcination, and investigated the influence of calcination temperature and time on the morphology, structure, and electrochemical properties of LaCoO_3_ nanomaterials. Then, based on the optimal electrochemical performance of LCO-700-4 electrode sample, the newly synthesized nanocomposites of Sr-doping (LSCO-0.2) and rGO-compounding (rGO@LCO) through rational design exhibited a 1.45-fold and 2.03-fold enhancement in its specific capacitance (specific capacity). The rGO@LCO electrode with better electrochemical performances was further explored by assembling rGO@LCO//rGO asymmetric supercapacitor system (ASS) with aqueous electrolyte. The result showed that the ASS delivers a high energy density of 17.62 W h kg^−1^ and an excellent cyclic stability with 94.48% of initial capacitance after 10,000 cycles, which are good electrochemical performances among aqueous electrolytes for green and new efficient energy storage devices.

## Introduction

With the depletion of fossil fuel reserves, the increase of energy cost, and the aggravation of environmental pollution, the research and development of efficient and reliable energy storage and conversion devices that can fully obtain and utilize renewable energy resources are facing great challenges and have attracted extensive attention [1–3]. Supercapacitors (SCs), also known as ultracapacitors (UCs), as a bridge connecting the huge difference between conventional capacitors and batteries, have attracted extensive research in recent decades for their unique advantages, such as higher energy density than conventional capacitors, higher power density, ultra-long service life, and environment friendly in comparison with batteries, as well as high safety and fast charge-discharge ability [4–6]. SCs can be divided into electrical double layer capacitors (EDLCs) and faraday capacitors according to the different reaction process and charge storage mechanism. The ion accumulation occurred at the interface between the electrode and electrolyte to store charge is EDLCs, which is a physical process and mainly uses various carbon electrode materials [7–11]. While the charge stored at the surface or subsurface of the electrode materials by a fast reversible faraday reaction or ion intercalation/de-intercalation is faraday capacitors, which is a chemical reaction process and its electrode materials mainly include transition metal oxides (hydroxides), nitrides, conductive polymers, and so on [12–17]. Since electrode material plays a crucial role in the electrochemical properties of SCs, while pure carbon materials usually have low energy density, many researches and efforts are focused on the electrode materials for faraday capacitors with high specific capacitance and high energy density [12, 18–20].

Recently, ABO_3_-type perovskite oxides, where A is lanthanide or alkaline earth element and B is transition metal, have been widely studied and applied in the field of energy storage as a kind of electrode material with exceptional electronic structure, excellent ionic conductivity, and thermal stability [21–24]. Che et al. [25] reports a novel anion-intercalated pseudocapacitive electrode of perovskite oxide LaNiO_3_ synthesized using sol-gel method, and it exhibits excellent electrochemical performances with a high specific capacitance of 478.7 F g^−1^ at 0.1 mV s^−1^ and a good cycling stability of reducing 5.5% charge-discharge efficiency after 15,000 cycles. Shafi et al. [26] synthetizes 3D polyhedron structured LaMnO_3_ nanoparticles using natural lemon juice (LJ) as a green surfactant, and the defined LMO nanoparticle shows a 3-fold enhancement in its specific capacitance. What Is more, the symmetric two electrode cell assembled with LMO/3.0 delivers a high energy density of 52.5 Wh kg^−1^ at a power density of 1000 W kg^−1^, and an outstanding cyclic stability with 97% retention of its maximum capacitance and 117% of its initial capacitance over 10,000 cycles. As the transition metal located in B position in ABO_3_-type perovskites, Mn, Ni, and Fe have been studied a lot for energy storage materials [22, 27–29]. Therefore, Co element with similar properties is also worthy of further study, especially for its modified nanocomposite [30–34]. However, the reported electrochemical properties of LaCoO_3_ and its modified composites are less than those of LaNiO_3_, LaMnO_3_, and other similar materials, which is worthy of further investigation.

In view of this, in this paper, we rationally designed and optimized the synthesis process, and successfully synthesized the perovskite-type LaCoO_3_ nanocomposite by sol-gel method followed by calcination, and investigated the influence of calcination temperature and time on the morphology, structure, and electrochemical properties of LaCoO_3_ nanomaterials. In addition, changing the lattice structure and oxygen vacancy of the material by doping and forming the composite material by combining with the material with high specific surface area are the two most commonly used techniques to enhance the electrochemical performance of a single pure phase electrode material. Therefore, based on the optimal LCO-700-4 electrode material, we choose two strategies of A-site element substitution (Sr) and composite with reduced graphene oxide (rGO) material with high specific surface area to further explore the influence of Sr-doping and rGO-compounding on its electrochemical performance of the newly synthesized LSCO-0.2 and rGO-compounding LaCoO_3_ (rGO@LCO) nanocomposites through rational design. Compared with pure LaCoO_3_ (LCO) electrode materials, the electrochemical properties of LSCO-0.2 and rGO@LCO nanocomposite electrodes have been significantly improved, especially for rGO@LCO electrode. The better electrochemical performance of the rGO@LCO electrode displays a high specific capacitance of 416 F g^−1^ (specific capacity: 63.56 mAh g^−1^) at a current density of 0.5 A g^−1^, and a good rate capability. When an asymmetric supercapacitor system (ASS) is assembled with rGO@LCO electrode as the positive electrode and rGO electrode as the negative electrode, the rGO@LCO//rGO ASS exhibits a high energy density of 17.62 W h kg^−1^ at a power density of 170 W kg^−1^, and an excellent cyclic stability with 94.48% of initial capacitance after 10,000 cycles at a large scan rate of 100 mV s^−1^. This result demonstrates that LaCoO_3_ and LaCoO_3_-based nanocomposites as electrode materials have great potential in green and efficient new energy storage devices.

## Experimental Methods

### Synthesis of Porous LaCoO_3_ and Sr-Doped LaCoO_3_ Nanomaterials

All chemical reagents were analytical grade and were not further purified before using in this experiment. In a typical synthesis process, 1 mM of lanthanum nitrate hexahydrate and 1 mM of cobalt nitrate hexahydrate were dissolved in 40 ml of *N*,*N*-dimethylformamide (DMF) and magnetically stirred for 2 h. Later, 0.45 g of polyvinylpyrrolidone (PVP-K30) was slowly added into the above mixture to get a homogeneous solution by continuous magnetic stirring for 3 h. And then, the homogeneous mixture was heated and stirred until the gel formed. Then the obtained gel was transferred to the crucible and placed in a muffle furnace for annealing treatment at 600 °C for 4 h at a heating rate of 5 °C min^−1^. The obtained sample naturally cooled to room temperature was LaCoO_3_-600 °C-4 h (recorded as LCO-600-4) nanomaterials. Under the same synthetic process, a series of LCO_S_ nanomaterials were obtained by controlling the reaction temperature and time, and recorded as LCO-700-4, LCO-800-4, LCO-700-2, and LCO-700-3.

The synthesis process of Sr-doped LaCoO_3_ nanocomposites was similar to the above-mentioned LCOs materials, except that in the initial step: 1 mM of lanthanum nitrate hexahydrate was changed to 0.8 mM of lanthanum nitrate hexahydrate and 0.2 mM of strontium nitrate, and the subsequent process was exactly the same. The synthesized Sr-doped LCO-700-4 nanocomposite was La_0.8_Sr_0.2_CoO_3-δ_ and recorded as LSCO-0.2.

### Synthesis of Porous rGO@LaCoO_3-δ_ Nanomaterials

Graphene oxide (GO) was prepared from natural flake graphite powder by a modified Hummer’s method [15]. In a simple solid-state method followed by high-temperature heat treatment synthesis process, 10 mg of prepared GO was grinded into powder in a mortar. Then 90 mg of synthesized LCO-700-4 sample was added into it and continue to grind them. After two materials were fully ground and mixed evenly, the mixture was transferred to a crucible and placed in the quartz tube of the tubular furnace. The heat treatment condition was 700 °C for 1 h under Ar atmosphere. After natural cooling to room temperature and collecting the materials, the rGO@LaCoO_3-δ_-700-4 nanocomposite was obtained, and recorded as rGO@LCO.

### Materials Characterization

The phase identification of the prepared samples were performed on the powder X-ray diffraction (Bruker D8 Advance) pattern with Cu Kα irradiation (*λ* = 0.154056 nm) at a scanning rate of 0.2°s^−1^. The microstructure and morphology of the samples were investigated by a scanning electron microscope (SEM; Quanta 250 FEG, USA) and transmission electron microscope (TEM, JEOL JEM-2100). The specific surface area obtained from the results of nitrogen adsorption-desorption isotherms at 77 K used the multipoint Brunauer-Emmett-Teller (BET) method (ASAP 2020 analyzer, Micromeritics, USA). Before the measurements, the samples were degassed in vacuum at 150 °C for 6 h. The pore size distributions and pore volume data of samples were calculated from the desorption branch based on the Barrett-Joyner-Halenda (BJH) equation. X-ray photoelectron spectroscopy (XPS) performed with a Thermo Scientific ESCALAB 250 Xi using Al Ka radiation (USA) was to examine the surface states of samples. Raman spectrum is obtained using a Renishaw inVia Raman microscope employing a 532 nm laser excitation (25% laser power).

### Electrochemical Measurements

The electrochemical performances of these electrodes were tested using a conventional three-electrode system and two-electrode system in 6 M KOH electrolyte, which performed on a CHI660E electrochemical workstation (Shanghai Chenhua, China) at room temperature. In this three-electrode system, the prepared electrode, a platinum plate electrode, and saturated calomel electrode (SCE) were served as working electrode, counter electrode, and reference electrode, respectively. The fabrication process of working electrode as follows: the prepared sample (80 wt%), conductive carbon black (10 wt%), and polytetrafluoroethylene (PTFE, 10 wt%) binder were mixed thoroughly by adding isopropyl alcohol. After mixing to form a homogeneous phase, the slurries were coated onto the nickel foam and pressed together with roller press. Afterwards, the fabricated electrode was trimmed carefully and dried overnight in a vacuum oven at 75 °C. The load of each electrode was about 1.8 mg.

The electrochemical performance measurements included cyclic voltammetry (CV), galvanostatic charge/discharge (GCD), and electrochemical impedance spectroscopy (EIS). The working electrode was immersed in the electrolyte overnight before test to promote the full contact between the electrolyte and the active material of working electrode. The gravimetric specific capacitances of the electrode were calculated based on the Eq. (1):

1 $$ Sc=\frac{I\times \Delta  t}{m\times \Delta  V} $$

where *Sc* (F g^−1^) is the specific capacitances, Δ*t* (s) is the discharge time, *m* (g) is the mass of active material, and Δ*V* is the range of potential window.

As for the two-electrode system, an asymmetric supercapacitor system (ASS) based on rGO@LCO electrode as the positive electrode and the rGO electrode as the negative electrode. And the mass of both electrodes was calculated according to the Eq. (2). Furthermore, the specific capacitance (*C*, F g^−1^), energy density (*E*, W h kg^−1^) and power density (*P*, kW kg^−1^) of the ASS were calculated based on the Eqs. (3), (4), and (5), respectively:

2 $$ \frac{m_{+}}{m_{-}}=\frac{Sc_{-}\times {\Delta  V}_{-}}{Sc_{+}\times {\Delta  V}_{+}} $$

3 $$ C=\frac{I\Delta  t}{M\Delta  V}\kern0.5em $$

4 $$ E=\frac{1}{2\times 3.6}C{\left(\Delta  V\right)}^2\kern0.5em $$

5 $$ P=\frac{3600E}{\Delta  t} $$

where *m*_*+*_ (g) and *m*_−_ (g) are the mass of positive electrode and negative electrode, respectively. *Sc*_*+*_ (F g^−1^) and *Sc*_−_ (F g^−1^) is the specific capacitance of positive electrode and negative electrode respectively calculated according to the three-electrode system. *M* (g) is the total mass of both electrodes.

## Results and Discussion

### Effects of Calcination Temperature and Time on the Morphology, Structure, and Electrochemical Properties of LCOs Materials

**Fig. 1:**
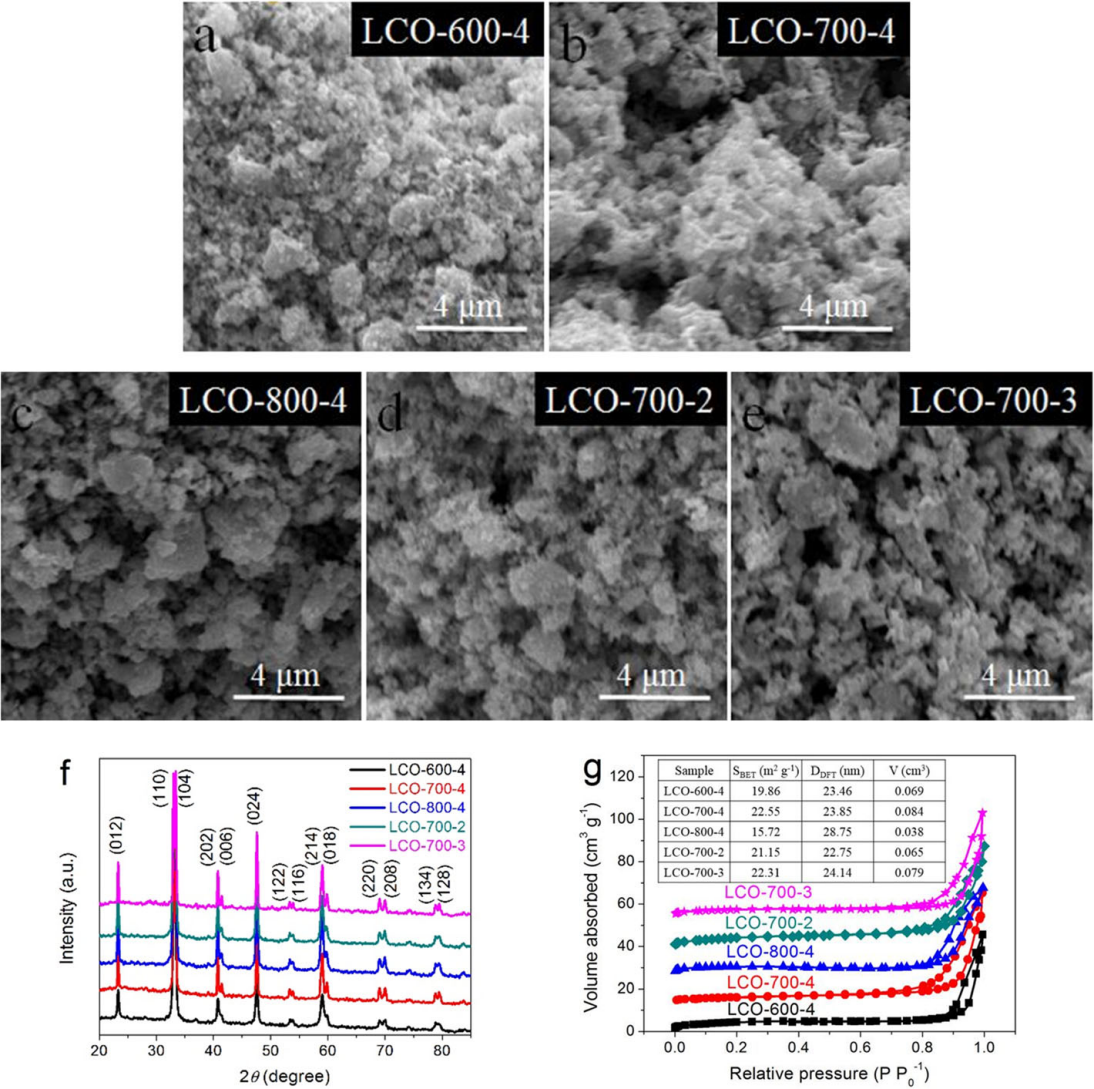
**a**–**e** SEM images, **f** XRD patterns, and **g** N_2_ adsorption-desorption isotherms of the as-prepared LCOs samples at different calcination temperatures and times

The scanning electron microscopy (SEM) images of LCOs nanomaterials prepared at different calcination temperatures and times are presented in Fig. 1a–e. It can be seen that the morphologies of all samples prepared under different calcining conditions are generally similar, and the subtle differences are mainly reflected in the particle size and porosity. With the increase of calcining temperature, the obtained LCO materials have more loose and abundant pore structure, but excessive temperature (Fig. 1c) will make the small particles to form a compact and large block structure, which reduces the porosity of the LCO material. And with the same calcination temperature and different calcination times, the LCO material shows a very similar morphology. Figure 1f shows the X-ray power diffraction (XRD) patterns of all prepared LCOs materials. The strong diffraction peaks at 2*θ* = 23.3°, 32.9°, 33.3°, 40.7°, 47.5°, and 59.0° are successfully indexed to the (012), (110), (104), (202), (024), and (214) crystal planes, respectively, which is attributed to the facets of hexagonal LaCoO_3_ according to JCPDS no: 48-0123. It can be seen that all LCOs samples have same diffraction peaks and no impurity peak, confirming a high purity of the samples. In addition, by careful comparison, it can be seen that compared with calcination time, the difference of calcining temperature has a greater impact on the crystallinity of materials. And the higher the temperature, the higher the crystallinity for the prepared LCOs samples. According to several main diffraction peaks of LCO samples and calculated according to the Scherer’s formula, the average crystalline size of LCO-700-4 sample is about 44.87 nm, which is slightly different from other samples at different calcining conditions (LCO-600-4 (43.65 nm), LCO-800-4 (47.15 nm), LCO-700-2 (44.53 nm), LCO-700-3 (44.15 nm)). A typical type-IV isotherm in the N_2_ adsorption-desorption isotherms indicates the existence of the mesoporous structure in all LCOs samples (Fig. 1g). In addition, it can be seen from the inset (Table 1) in Fig. 1g that excessive calcination temperature will seriously affect the specific surface area and porosity of the material.

**Table 1:** Textural parameters of the LCO-700-4, LSCO-0.2 and rGO@LCO samples

Sample	*S*_BET_ (m^2^ g^−1^)	*D*_DFT_ (nm)	*V* (cm^3^ g^−1^)
LCO-700-4	22.55	23.85	0.084
LSCO-0.2	29.74	25.21	0.121
rGO@LCO	59.89.	18.79	0.204

*S*_BET_ represents the BET surface area; *D*_DFT_ is the DFT desorption average pore diameter; *V* represents the total pore volume

**Fig. 2:**
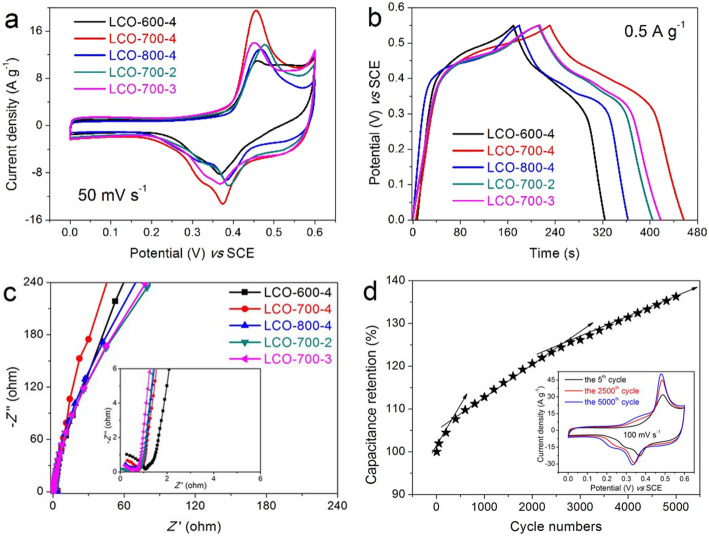
**a** CV curves at 50 mV s^−1^, **b** GCD curves at 0.5 A g^−1^, and **c** Nyquist plots in the frequency range of 100 kHz to 0.01 Hz for all prepared LCOs electrodes at different calcination temperatures and times. **d** Cycling stability of LCO-700-4 electrode at 100 mV s^−1^ for 5000 cycles (insert shows the comparison of CV curves at 5th cycle, 2500th cycle, and 5000th cycle)

Figure 2a shows the CV curves of all electrodes at a scanning rate of 50 mV s^−1^. It can be seen clearly that the reaction process includes the characteristics of double electric layer process (quasi rectangle in low potential area) and faraday reaction (obvious oxidation-reduction peaks), indicating that the generated capacitance includes double electric layer capacitance and faraday capacitance. Moreover, by comparison, it can be concluded that the area enclosed by CV curve of LCO-700-4 electrode is larger, which means that it has larger capacitance. The corresponding GCD curves of these electrodes at 0.5 A g^−1^ are displayed in Fig. 2b. The GCD curves with the nonlinear triangles further show that the capacitance generated in the charging-discharging process includes the double layer capacitance and the faraday capacitance. And calculated from GCD curves, the specific capacitance of LCO-700-4 electrode is 205.04 F g^−1^ (specific capacity: 31.33 mAh g^−1^), which is slightly higher than that of LCO-600-4 electrode (140.03 F g^−1^, 21.39 mAh g^−1^), LCO-800-4 electrode (166.23 F g^−1^, 25.40 mAh g^−1^), LCO-700-2 electrode (174.37 F g^−1^, 26.64 mAh g^−1^), and LCO-700-3 electrode (185.22 F g^−1^, 28.30 mAh g^−1^), respectively.

The impedance behavior of these LCOs electrodes are measured in the frequency range of 100 kHz to 0.01 Hz with an amplitude of 5 mV to understand the charge transfer process. As shown in Fig. 2c, all Nyquist plots contain an arc in the high frequency and an approximate straight line with a high slope in the low frequency. The diameter of the distorted arc in the high-frequency region represents the charge transfer resistance (*R*ct). It can be seen from the insert in Fig. 2c that all LCOs electrodes have very small *R*ct except for LCO-600-4 electrode with a slightly larger *R*ct, indicating that the perovskite LaCoO_3_ material is very favorable for the rapid transmission of charge [35, 36]. The slope of the straight line in the low-frequency region represents Warburg impedance (*W*o), which mainly reflects the diffusion resistance of electrolyte and proton in the active material [24, 37]. A larger slope means a lower *W*o. Therefore, it can be seen that the LCO-700-4 electrode material has a better diffusion dynamics properties of electrolyte and proton.

The cycling stability of LCO-700-4 electrode with the optimal electrochemical performance for 5000 cycles at a large scanning rate of 100 mV s^−1^ is presented in Fig. 2d. It can be observed that the capacitance increases with cycle numbers. And with further careful observation, the capacitance increases faster as the number of cycles increases, especially in the initial 800 cycles. Then after about 3000 cycles, the increment of capacitance decreases gradually as the number of cycle increases, which can be seen from the decrease in the approximate slope of the capacitance retention curve in Fig. 2d. Therefore, we infer that the LaCoO_3_ nanomaterial has a stable structure and can buffer the volume change under a large scanning rate, so that the LaCoO_3_ nanomaterial is activated continuously in the early stage of redox reaction until it is fully activated. In the later stage, with the continuous sufficient infiltration of electrolyte and active substance, the activation of internal active sites is further promoted, thus further enhancing the specific capacitance [24, 38]. The increase of capacitance is fully illustrated by the CV curves at the 5th, 2500th, and 5000th cycles as shown in the insert in Fig. 2d, which can be seen that the area enclosed by CV curve increases with the increase of cycle numbers.

### Effects of Sr-Doping and rGO-Compounding on the Morphology, Structure, and Electrochemical Properties of the Newly Synthesized Nanocomposites

**Fig. 3:**
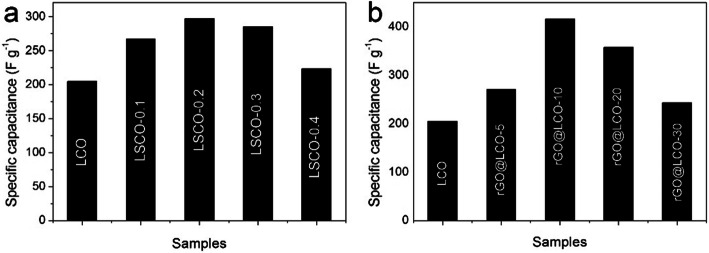
The specific capacitance of **a** LSCOs, and **b** rGO@LCOs electrodes at 0.5 A g^−1^

Through reasonable adjustment and control of Sr-doping amount and rGO-compounding content, we further explore the influence of Sr-doping and rGO-compounding on the newly synthesized LCO-700-4-based nanocomposites, in order to obtain better performance of the LCO-based composite electrode. The specific capacitance of Sr-doping LCO series electrodes are showed in Fig. 3a. With the increase of Sr-doping content, the specific capacitance of Sr-doping LaCoO_3_ (LSCOs) composite electrode increases first and then decreases, and the LSCO-0.2 electrode (La_1-x_Sr_x_CoO_3_, *x* = 0.2) shows the best capacitance performance. Simultaneously, the specific capacitance of rGO-compounding LCO series electrodes are presented in Fig. 3b. It can be seen that rGO@LCO-10 (LCO: 90 mg, rGO: 5 mg, 10 mg, 20 mg, 30 mg) electrode has the best capacitance property. However, the excess of rGO in composites makes the specific capacitance decrease sharply. We speculate that the agglomeration caused by excessive rGO not only fails to effectively increase the capacitance, but also affects the pore structure of the composite, which is not conducive to the ion/electron transport, thus reducing the electrochemical performance of the rGO@LCO composites. In order to further explore the influence of Sr-doping and rGO-compounding on the electrochemical performance of the newly synthesized LCO-700-4-based nanocomposites, and make a comparative analysis with LCO-700-4 electrode material. Therefore, in the following research of this paper, we focus on exploring the electrode materials with the optimal capacitance property in their respective series to compare with LCO materials.

**Fig. 4:**
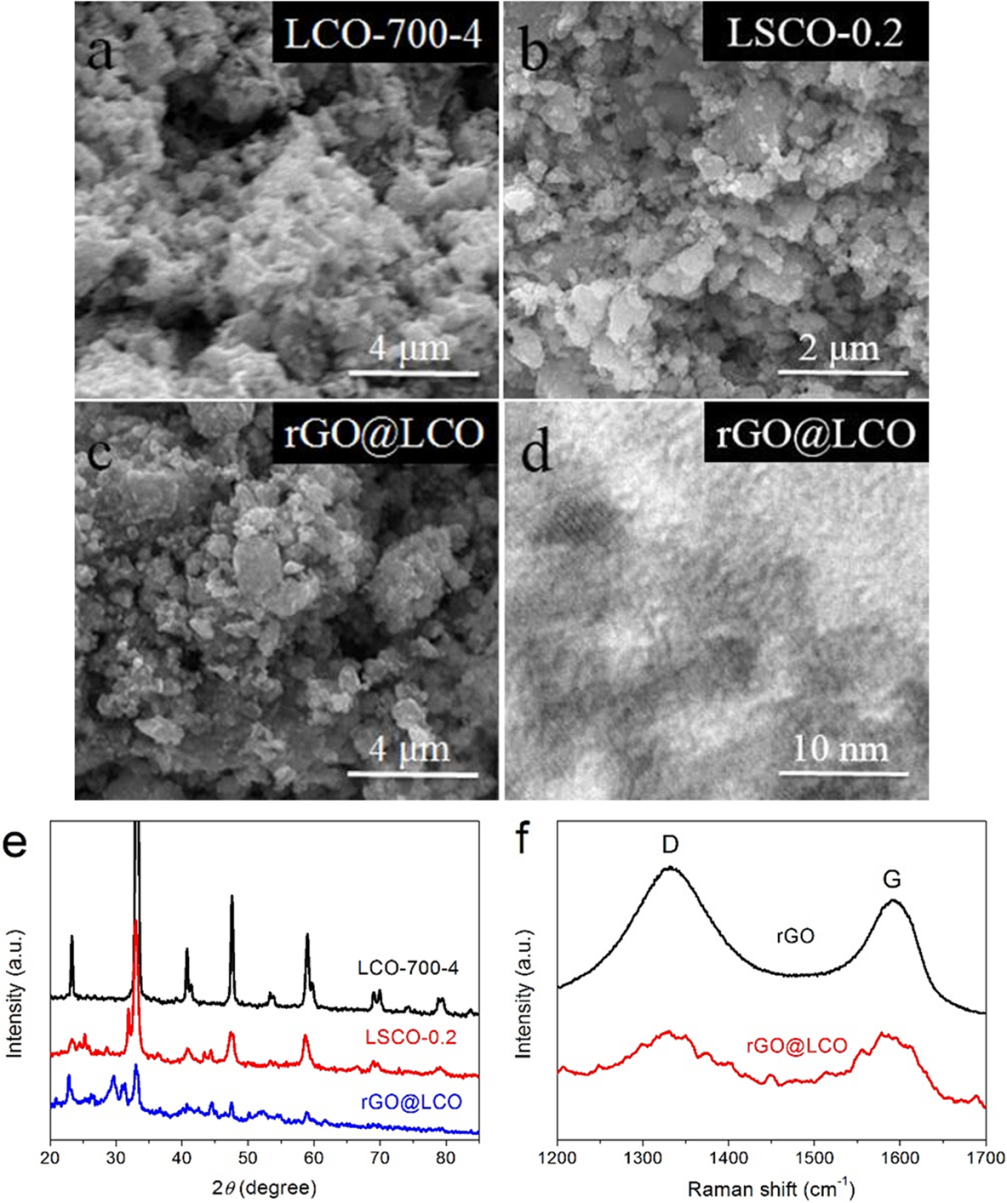
**a**–**c** SEM images of the LCO-700-4, LSCO-0.2, and rGO@LCO samples. **d** HRTEM image of rGO@LCO composite. **e** XRD patterns of the LCO-700-4, LSCO-0.2, and rGO@LCO samples. **f** Raman spectra of rGO and rGO@LCO samples

The morphology and microstructure of LCO-700-4, LSCO-0.2, and rGO@LCO samples are presented in Fig. 4a–c. It can be observed that the LSCO-0.2 and rGO@LCO nanocomposites have more porous structure and smaller particle morphology with more evenly dispersed. Furthermore, it also can be observed that the LCO material can be well dispersed in and between the rGO sheets, effectively reducing the occurrence of agglomerated graphene with the property of easy agglomeration. The detailed structure of rGO@LCO composite is further characterized by HRTEM image, as shown in Fig. 4d. The porous structure of LCO materials can be well dispersed in rGO materials. In addition, due to the influence of GO reactants, it can be seen that the lattice fringes of the LCO materials are not obvious, which indicates that the crystallinity of the LCO materials in rGO@LCO composites is reduced by the influence of GO materials. The crystal structure and phase composition of the synthesized LSCO-0.2 and rGO@LCO are characterized by XRD, and the comparison of XRD pattern for their and LCO-700-4 sample is displayed in Fig. 4e. It can be seen that the position of the main diffraction peak of LSCO-0.2 sample is basically the same as that of LCO-700-4 sample. But due to the influence of Sr-doping, the intensity of diffraction peak decreases and a small number of small peaks appear. Similarly, the major diffraction peaks of LCO-700-4 samples are also reflected in rGO@LCO nanocomposites. However, at the same time, due to the high temperature reaction between LaCoO_3_ and rGO materials, a small amount of by-product of LaCO_3_OH materials (JCPDS no: 26-0815) appears in the rGO@LCO nanocomposites, corresponding to the diffraction peak of the position located at about 2*θ* = 29.8°. In addition, the weak peak located at around 2*θ* = 26.2° should be considered to be the diffraction peak of rGO materials. Figure 4f displays the Raman spectra of rGO and rGO@LCO samples. The characteristic peak located at around 1331.17 cm^−1^ corresponds to the D-band, which mainly represents the defect and the disordered structures of graphene. And the characteristic peak located at around 1594.53 cm^−1^ corresponds to the G-band, which mainly produced by the in-plane stretching vibration of sp^2^ carbon atom. In addition, a slight decrease of the *I*_D_/*I*_G_ value from rGO to rGO@LCO indicated fewer defects in the influence of LCO sample participating in the reaction.

**Fig. 5:**
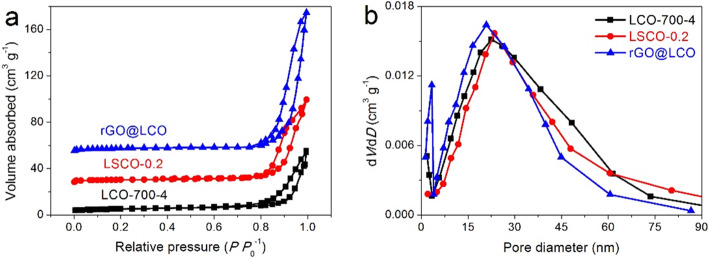
**a** Nitrogen adsorption-desorption isotherms and **b** the corresponding pore size distribution curves of LCO-700-4, LSCO-0.2, and rGO@LCO samples

The nitrogen adsorption-desorption isotherms of LCO-700-4, LSCO-0.2, and rGO@LCO samples illustrated in Fig. 5a display a typical type-IV isotherm with a hysteresis loop at *P P*_0_^−1^ of about 0.75, indicating the existence of a large number of mesopore nanostructures. Simultaneously, the capillary condensation occurs at higher pressure also means that these samples have both mesopores and macropores [39]. The corresponding pore size distribution curves are presented in Fig. 5b. It can be observed that the pore size of these materials is mainly concentrated in 10–50 nm. However, due to the existence of rGO materials, there are more pores with diameter less than 5 nm in rGO@LCO composite. The specific surface areas (*S*_BET_) of LCO-700-4, LSCO-0.2, and rGO@LCO are calculated to be 22.55, 29.74, and 59.89 m^2^ g^−1^, respectively (Table 1). The rGO@LCO with the largest *S*_BET_ is mainly attributed to the high specific surface area characteristics of rGO nanosheets. Due to the influence of S-doping and rGO-compounding, the *S*_BET_ and pore volume of the synthesized LSCO-0.2 and rGO@LCO are increased to some extent compared with the pure LCO-700-4 material, which will increases the reaction sites and charge storage sites, thus improving the capacitance of the composites.

**Fig. 6:**
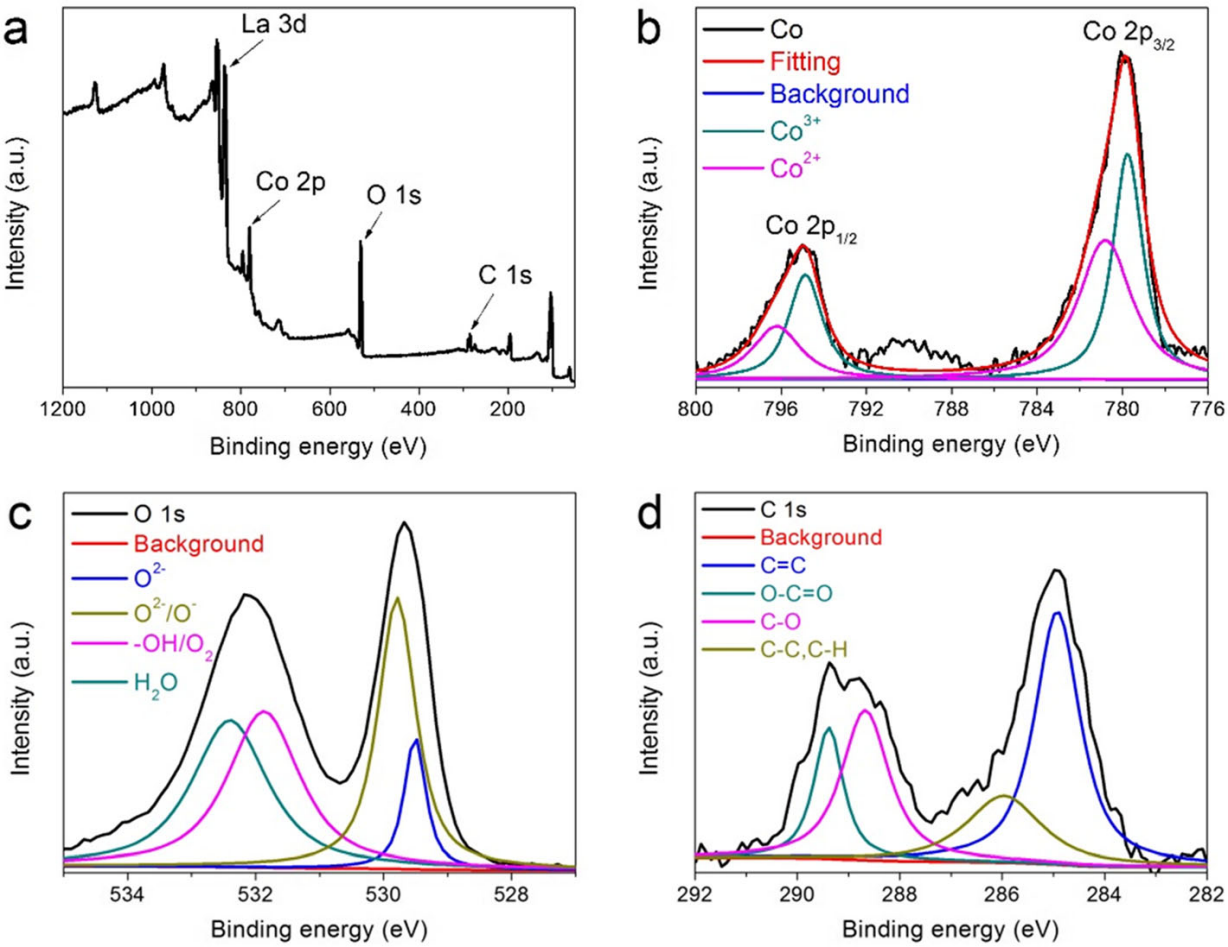
**a** XPS survey scan of rGO@LCO surface, and high-resolution spectrum of **b** Co 2p, **c** O 1s, and **d** C 1s

The surface properties of the synthesized rGO@LCO nanocomposite are confirmed by XRS. As presented in Fig. 6a, the full survey scan XPS spectrum demonstrates the presence of La, Co, O, and C elementals. Deconvoluted spectrum of Co 2p presented in Fig. 6b indicates the existence of Co elemental in two oxidation of Co^2+^ and Co^3+^. And the binding energies at 779.7 and 794.8 eV are attributed to Co^3+^, while the binding energies at 780.8 and 796.2 eV are ascribed to Co^2+^ [40, 41]. The high-resolution spectrum of O 1s showed in Fig. 6c exhibits four peaks at 532.4, 531.8, 529.8, and 529.5 eV after deconvolution of O 1s, corresponding to surface adsorbed H_2_O, surface adsorbed oxygen or hydroxyl groups, highly oxidative oxygen species, and surface lattice oxygen, respectively [24, 41, 42]. It is believed that the higher of oxygen vacancies in perovskite oxides is favorable for the adsorption capacity of OH^-^, thus accelerating the kinetics of surface oxidation-reduction reaction and improving the conductivity and electrochemical performance [24, 43]. The high-resolution spectrum of C 1s illustrated in Fig. 6d are mainly consisted of four peaks, and the binding energies at 289.4, 288.7, 286.0, and 284.8 eV are corresponding to the groups of O-C=O, C-O, C-C and C-H, and C=C, respectively [15]. These results are consistent with the previous SEM and XRD results, which strongly proves the existence of rGO and LaCoO_3_ materials.

**Fig. 7:**
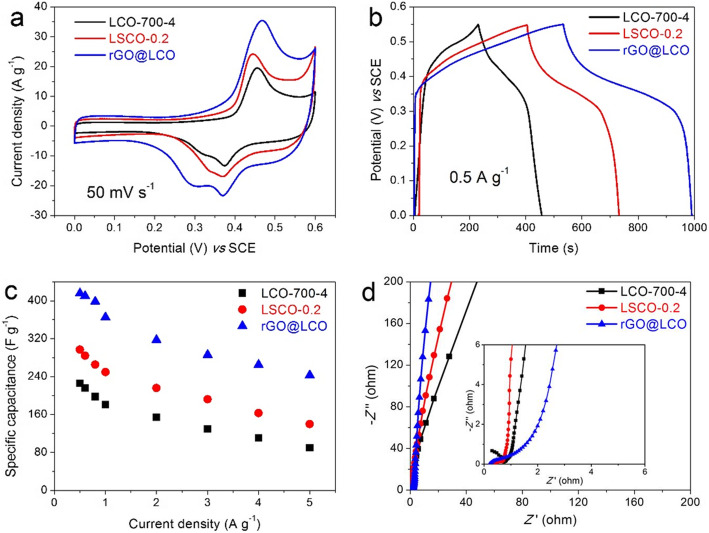
**a** CV curves at 50 mV s^−1^, **b** GCD curves at 0.5 A g^−1^, **c** rate capability, and **d** Nyquist plots in the frequency range of 100 kHz to 0.01 Hz for LCO-700-4, LSCO-0.2, and rGO@LCO electrodes

The effects of Sr-doping and rGO-compounding on the electrochemical properties of the newly synthesized nanocomposites are illustrated in Fig. 7. The CV curves of LCO-700-4, LSCO-0.2, and rGO@LCO electrodes at a scan rate of 50 mV s^−1^ are illustrated in Fig. 7a. It can be seen that the shapes of CV curve have not change much, and the areas enclosed by CV curve of LSCO-0.2 electrode and rGO@LCO are significantly larger than that of LCO-700-4 electrode, which means that the capacitance is improved with Sr-doping or rGO-compounding. After calculation based on the GCD curves (Fig. 7b), the capacitance of LSCO-0.2 electrode and rGO@LCO electrode are 297.09 F g^−1^ (specific capacity: 45.39 mAh g^−1^) and 416 F g^−1^ (63.56 mAh g^−1^) at a current density of 0.5 A g^−1^, which are 1.45 times and 2.03 times of that of LCO-700-4 electrode, respectively. The equation of specific capacitance changing with current density of LCO-700-4, LSCO-0.2, and rGO@LCO electrodes are illustrated in Fig. 7c. When the current increased by 10 times (from 0.5 to 5 A g^−1^), the capacitance of LSCO-0.2 electrode and rGO@LCO electrode remained 47.01% and 58.40%, which is higher than that of LCO-700-4 electrode (39.71%), indicating that the rate capability of LSCO-0.2 electrode and rGO@LCO electrode is significantly improved. The comparison of Nyquist plots for LCO-700-4, LSCO-0.2, and rGO@LCO electrode is displayed in Fig. 7d. After careful observation and comparison, it can be seen that the values of *R*ct and *W*o for three electrodes are as follows: *R*ct (LSCO-0.2) < *R*ct (rGO@LCO) < *R*ct (LCO-700-4), *W*o (rGO@LCO) < *W*o (LSCO-0.2) < *W*o (LCO-700-4), which shows that the nanocomposites have better conductivity and ion diffusion dynamics than the pure LCO-700-4 material. These results show that the new nanocomposites obtained by Sr-doping or rGO-compounding can greatly enhance the electrochemical performance, especially for the rGO@LCO nanocomposites. Therefore, based on rGO@LCO nanocomposites as positive material, we will then assemble it into an asymmetric two-electrode system for further research.

**Fig. 8:**
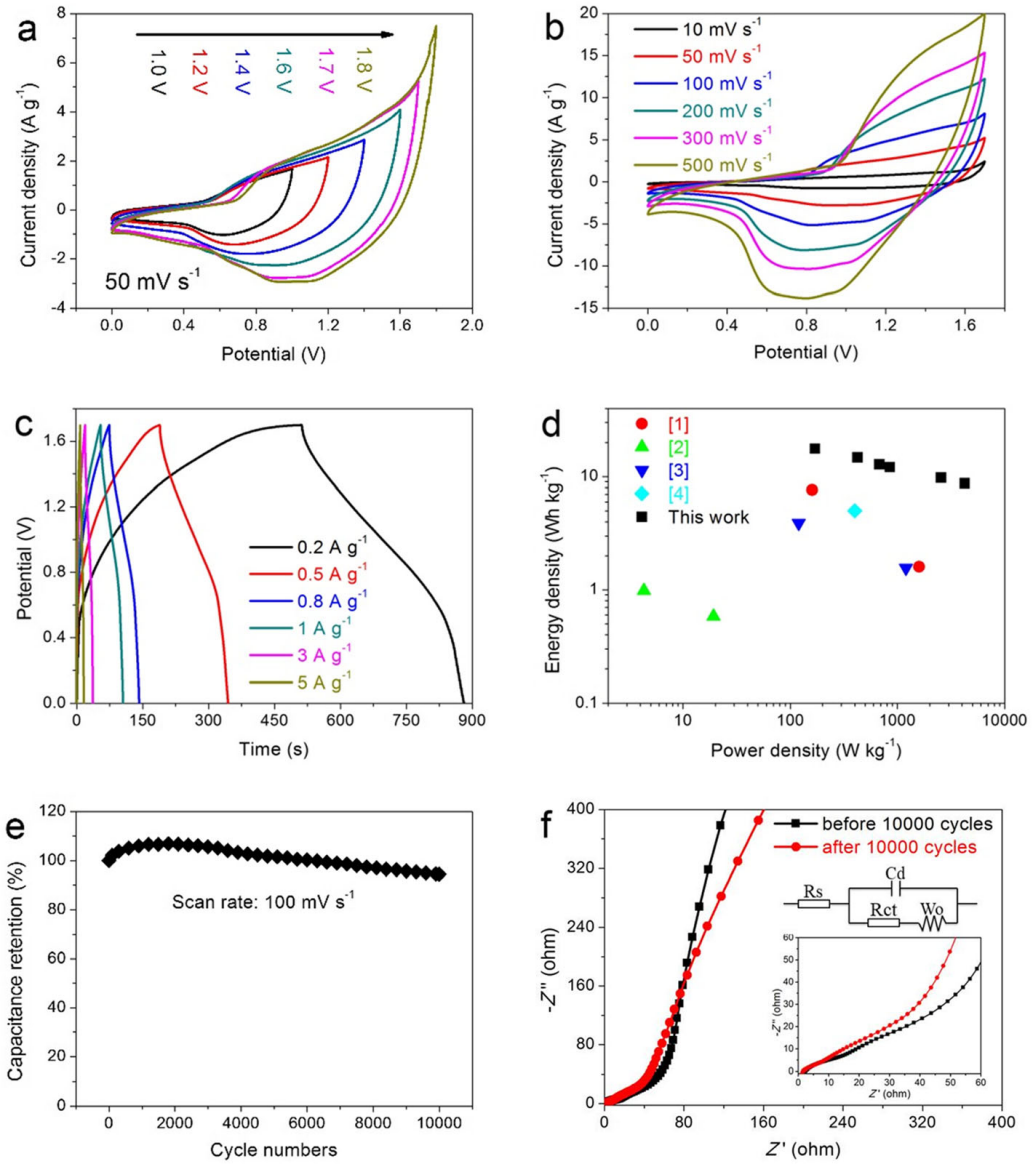
Electrochemical performances of rGO@LCO//rGO asymmetric supercapacitor system (ASS) in 6 M KOH electrolyte: **a** CV curves at various potential windows from 0–1.0 to 0–1.8 V with a scan rate of 50 mV s^−1^. **b** CV curves. **c** GCD curves. **d** Ragone plot. **e** Cycle stability at 100 mV s^−1^ for 10,000 cycles. **f** Nyquist plots of rGO@LCO//rGO ASS before and after 10000 cycles

An assembled asymmetric supercapacitor system (ASS) using the rGO@LCO as positive electrode and the rGO as negative electrode to explore the rGO@LCO nanocomposite as an efficient energy storage electrode material in the practical application. A comparative CV curves of different potential window ranges from 0–1 to 0–1.8 V at 50 mV s^−1^ are presented in Fig. 8a. It can be clearly observed that 0–1.7 V is the optimal voltage window selection, which is judged from the fact that 0–1.7 V is a stable voltage window and can avoid polarization phenomenon. Therefore, the CV curves and GCD curves based on the optimal voltage window are displayed in Fig. 8b, c, respectively. The oxidation-reduction peaks of CV curves and the asymmetric triangles of GCD curves confirm the formation of fine EDLC and faraday capacitance in rGO@LCO//rGO ASS. In addition, even if the scanning rate increases from 10 to 500 mV s^−1^, the CV curves still maintain a similar shape, showing excellent characteristics of large current charging and discharging. Moreover, the less obvious *IR* drop on the GCD curves indicates that the electrode material has a very small resistance [44]. The energy and power densities of the rGO@LCO//rGO ASS derived from the GCD curves are also estimated and the results in the form of Ragone plot is displayed in Fig. 8d. Calculated by Eqs. (4) and (5), the rGO@LCO//rGO ASS delivers a high energy density of 17.62 W h kg^−1^ at a power density of 170 W kg^−1^, which is mainly due to the improvement of specific capacitance and the extended voltage window. And even at power density as high as 4250 W kg^−1^, the ASS still delivers a high energy density of 8.73 W h kg^−1^, which is an attractive result and competitive compared to previous reports [45–48].

The ultra-long cycle stability is another important performance index of new energy storage devices. Therefore, we have conducted 10,000 cycle tests on rGO@LCO//rGO ASS at a large scanning rate of 100 mV s^−1^, and the analysis result is presented in Fig. 8e. It can be seen that the specific capacitance of the first 2000 cycles increases gradually with the increase of the cycle numbers, up to 106.82% of the initial capacitance. This is mainly attributed to the continuous full penetration of the electrolyte, which promotes the activation of the internal reaction site more fully, thus resulting in the improvement of the capacitance. And with the number of cycles further increased to 10,000, the rGO@LCO//rGO ASS still retains 94.48% of the initial capacitance, showing excellent ultra-long cycle stability, which also means that the electrode material still has a stable nanostructure and is favorable for ion/electron transport under the condition of large current charge-discharge. Figure 8f shows the Nyquist plots of rGO@LCO//rGO ASS before and after 10,000 cycles. In contrast, the rGO@LCO//rGO ASS shows a smaller *R*ct and a slightly increased *W*o after 10,000 cycles, which further demonstrates the stable nanostructure of the electrode material. The above results show that the synthesized rGO@LCO nanomaterial electrode exhibits attractive electrochemical performance, which is comparable and superior to those previously reported literature in many cases (Table 2).

**Table 2:** Comparison of electrochemical performances for some composite electrodes in the previous literatures

Samples	*Sc* (F g^−1^)	Current density (scan rate)	Electrolyte	*E* (W h kg^−1^) *P* (W kg^−1^)	Capacitance retention (cycle numbers)	Ref.
ZnFe_2_O_4_/NRG	244	0.5 A g^−1^	1 M KOH	6.7; 3000	84.4% (1000)	[49]
Y_2_NiMnO_6_	77.76	30 A g^−1^	0.5 M KOH	0.89; 4.32	70.17% (> 1800)	[46]
La_0.85_Sr_0.15_MnO_3_	198	0.5 A g^−1^	1 M KOH	//	~ 79% (1000)	[37]
La_0.8_Na_0.2_Fe_0.8_ Mn_0.2_O_3_	56.4	3 mV s^−1^	1 M H_2_SO_4_	14.39; /	86% (1000)	[29]
MOF-derived Co(OH)_2_	604.5	0.1 A g^−1^	6 M KOH	13.6; 140	80% (2000)	[50]
La_1-x_Ca_x_MnO_3_	170	1 A g^−1^	1 M KOH	7.6; 160	7.7% (2000)	[45]
Ag/LSC	517.5	1 mA cm^−2^	1 M KOH	21.9 mW h m^-3^; 90.1 mW m^-3^	81.2% (3000)	[51]
LaSr_3_Fe_3_O_10-δ_	380	0.1 A g^−1^	6 M KOH	/; /	87.1% (1000)	[52]
(La_0.75_Sr_0.25_)_0.95_MnO_3-δ_	56	2 mV s^−1^	1 M Na_2_SO_4_	/; /	98% (1000)	[39]
CeO_2_ mixed LaMnO_3_	262	1 A g^−1^	1 M Na_2_SO_4_	17.2; 1015	98% (2000)	[53]
rGO@LCO	416	0.5 A g^−1^	6 M KOH	17.62 (170); 8.73 (4250)	94.48% (10,000)	This work

## Conclusions

In summary, we successfully synthesized the perovskite LaCoO_3_ nanomaterials by a simple and usual sol-gel method followed by calcination, which is applied to electrode materials for supercapacitors and explored the influence of calcination temperature and time on their electrochemical properties. The results showed that the calcination temperature has a greater influence on the electrochemical properties than calcination time. Based on the optimal electrochemical properties of the LCO-700-4 electrode materials, the LSCO-0.2 and rGO@LCO nanocomposites were successfully synthesized by rational design. The results of the investigation of the electrochemical performance for these newly synthesized nanocomposites showed that the specific capacitance, rate capability, and conductivity of LSCO-0.2 and rGO@LCO are significantly enhanced, with the specific capacitance being 1.45 and 2.03 times of that of LCO-700-4 electrode, respectively. The practical performance of rGO@LCO composite electrode was further studied by assembling an asymmetric supercapacitor system (ASS) of aqueous electrolyte using rGO@LCO as the positive electrode and rGO as the negative electrode. The test results showed that the rGO@LCO//rGO ASS exhibits excellent energy and power density, as well as an outstanding cyclic stability with 94.48% of initial capacitance after 10,000 cycles. As a potential energy storage electrode material, LaCoO_3_ and LaCoO_3_-based nanocomposites electrode with excellent electrochemical properties was worthy of further exploration, so as to make more breakthroughs and move toward practical application in green and efficient new energy storage devices.

## Data Availability

All data are fully available without restriction.

## Abbreviations

GO: Graphene oxide
rGO: Reduced graphene oxide
LCO: LaCoO_3_
LSCO: Sr-doping LaCoO_3_
rGO@LCO: rGO-compounding LaCoO_3_
ASS: Asymmetric supercapacitor system
SCs: Supercapacitors
UCs: Ultracapacitors
EDLCs: Electrical double layer capacitors
DMF: *N*,*N*-Dimethylformamide
PVP-K30: Polyvinylpyrrolidone
XRD: X-ray power diffraction
SEM: Scanning electron microscope
TEM: Transmission electron microscopy
BET: Brunauer-Emmett-Teller
BJH: Barrett-Joyner-Halenda
XPS: X-ray photoelectron spectroscopy
SCE: Saturated calomel electrode
PTFE: Polytetrafluoroethylene
CV: Cyclic voltammetry
GCD: Galvanostatic charge/discharge
EIS: Electrochemical impedance spectroscopy
*R*ct: Charge transfer resistance
*W*o: Warburg impedance
HRTEM: High-resolution transmission electron microscopy
*S*_BET_: Specific surface areas
*D*_DFT_: DFT desorption average pore diameter

## References

[1] Bai XX, Wang Z, Luo JY, Wu WW, Liang YP, Tong X, Zhao ZH (2020). Hierarchical porous carbon with interconnected ordered pores from biowaste for high-perfromance supercapacitor electrodes. Nanoscale Res Lett.

[2] Wang FX, Wu XW, Yuan XH, Liu ZC, Zhang Y, Fu LJ, Zhu YS, Zhou QM, Wu YP, Huang W (2017). Latest advances in supercapacitors: from new electrode materials to novel device designs. Chem Soc Rev.

[3] Maurya DK, Murugadoss V, Angaiah S (2019). All-solid-state electrospun poly (vinylidene fluoride-co-hexafluoropropylene)/Li_7.1_La_3_Ba_0.05_Zr_1.95_O_12_ nanohybrid membrane electrolyte for high-energy Li-Ion capacitors. J Phys Chem C.

[4] Kirubasankar B, Murugadoss V, Lin J, Ding T, Dong MY, Liu H, Zhang JX, Li TX, Wang N, Guo ZH, Angaiah S (2018). In situ grown nickel selenide on graphene nanohybrid electrodes for high energy density asymmetric supercapacitors. Nanoscale.

[5] Zhang WY, Chen L, Liu HL, Kang HW, Zhang SR, Yang BC, Wang Y, Yuan MK, Li ZK (2020). High capacitive and super-long life supercapacitor fabricated by 7-aminoindole/reduced graphene oxide composite. Electrochim Acta.

[6] Raza W, Ali F, Raza N, Luo YW, Kim KH, Yang JH, Kumar S, Mehmood A, Kwon EE (2018). Recent advancements in supercapacitor technology. Nano Energy.

[7] Hwang JY, Li MP, El-Kady MF, Kaner RB (2017). Next-generation activated carbon supercapacitors: a simple step in electrode processing leads to remarkable gains in energy density. Adv Funct Mater.

[8] Li ZJ, Yang BC, Su YL, Wang HY, Groeper J (2015). Ultrafast growth of carbon nanotubes on graphene for capacitive energy storage. Nanotechnology.

[9] Liu TY, Zhang F, Song Y, Li Y (2017). Revitalizing carbon supercapacitor electrodes with hierarchical porous structures. J Mater Chem A.

[10] Sun JM, Li W, Lei E, Xu Z, Ma CH, Wu ZW, Liu SX (2019). Ultralight carbon aerogel with tubular structures and N-containing sandwich-like wall from kapok fibers for supercapacitor electrode materials. J Power Sources.

[11] Vijayakumar M, Santhosh R, Adduru J, Rao TN, Karthik M (2018). Activated carbon fibres as high performance supercapacitor electrodes with commercial level mass loading. Carbon.

[12] Jiang Q, Kurra N, Alhabeb M, Gogotsi Y, Alshareef HN (2018). All pseudocapacitive MXene-RuO_2_ asymmetric supercapacitors. Adv Energy Mater.

[13] Ke QQ, Guan C, Zhang X, Zheng MR, Zhang YW, Cai YQ, Zhang H, Wang J (2017). Surface-charge-mediated formation of H-TiO_2_@Ni(OH)_2_ heterostructures for high-performance supercapacitors. Adv Mater.

[14] Subasri A, Balakrishnan K, Nagarajan ER, Devadoss V, Subramania A (2018). Development of 2D La(OH)_3_/graphene nanohybrid by a facile solvothermal reduction process for high-performance supercapacitors. Electrochim Acta.

[15] Zhang WY, Kang HW, Liu HL, Yang BC, Liu YX, Yuan MK, Li ZK (2019). 1-Aminopyrene/rGO nanocomposites for electrochemical energy storage with improved performance. Chem Eng J.

[16] Kirubasankar B, Palanisamy P, Arunachalam S, Murugadoss V, Angaiah S (2019). 2D MoSe_2_-Ni(OH)_2_ nanohybrid as an efficient electrode material with high rate capability for asymmetric supercapacitor applications. Chem Eng J.

[17] Meng QF, Cai KF, Chen YX, Chen LD (2017). Research progress on conducting polymer based supercapacitor electrode materials. Nano Energy.

[18] Lukatskaya MR, Kota S, Lin ZF, Zhao MQ, Shpigel N, Levi MD, Halim J, Taberna PL, Barsoum MW, Simon P, Gogotsi Y (2017). Ultra-high-rate pseudocapacitive energy storage in two-dimensional transition metal carbides. Nat Energy.

[19] Kirubasankar B, Vijayan S, Angaiah S (2019). Sonochemical synthesis of a 2D-2D MoSe_2_/graphene nanohybrid electrode material for asymmetric supercapacitors. Sustainable Energy Fuels.

[20] Boota M, Chen C, Van Aken KL, Jiang JJ, GogotSI Y (2019). Organic-inorganic all-pseudocapacitive asymmetric energy storage devices. Nano Energy.

[21] Qian JM, Wang TT, Zhang ZM, Liu YG, Li JF, Gao DQ (2020). Engineered spin state in Ce doped LaCoO_3_ with enhanced electrocatalytic activity for rechargeable Zn-Air batteries. Nano Energy.

[22] Nan HS, Hu XY, Tian HW (2019). Recent advances in perovskite oxides for anion-intercalation supercapacitor: a review. Mat Sci Semicon Proc.

[23] Li ZJ, Zhang WY, Yuan CS, Su YL (2017). Controlled synthesis of perovskite lanthanum ferrite nanotubes with excellent electrochemical properties. RSC Adv.

[24] Lang XQ, Zhang HF, Xue X, Li CL, Sun XC, Liu ZT, Nan HS, Hu XY, Tian HW (2018). Rational design of La_0.85_Sr_0.15_MnO_3_@NiCo_2_O_4_ Core-Shell architecture supported on Ni foam for high performance supercapacitors. J Power Sources.

[25] Che W, Wei MR, Sang ZS, Ou YK, Liu YH, Liu JP (2018). Perovskite LaNiO_3-δ_ oxide as an anion-intercalated pseudocapacitor electrode. J Alloy Compd.

[26] Shafi PM, Joseph N, Thirumurugan A, Bose AC (2018). Enhanced electrochemical performances of agglomeration-free LaMnO_3_ perovskite nanoparticles and achieving high energy and power densities with symmetric supercapacitor design. Chem Eng J.

[27] Lv JB, Zhang YH, Lv Z, Huang XQ, Wang ZH, Zhu XB, Wei B (2016). Strontium doped lanthanum manganite/manganese dioxide composite electrode for supercapacitor with enhanced rate capability. Electrochim Acta.

[28] Li ZJ, Zhang WY, Wang HY, Yang BC (2017). Two-dimensional perovskite LaNiO_3_ nanosheets with hierarchical porous structure for high-rate capacitive energy storage. Electrochim Acta.

[29] Rai A, Thakur AK (2017). Effect of Na and Mn substitution in perovskite type LaFeO_3_ for storage device applications. Ionics.

[30] Cao Y, Lin BP, Sun Y, Yang H, Zhang XQ (2015). Symmetric/asymmetric supercapacitor based on the perovskite-type Lanthanum cobaltate nanofibers with Sr-substitution. Electrochim Acta.

[31] Guo GL, Ouyang K, Yu JP, Liu YH, Feng SL, Wei MR (2020). Facile synthesis of LaCoO_3_ with a high oxygen vacancy concentration by the plasma etching technique for high-performance oxygen ion intercalation pseudocapacitors. ACS Appl Energy Mater.

[32] Guo YZ, Shao TY, You HH, Li S, Li C, Zhang L (2017). Polyvinylpyrrolidone-assisted solvothermal synthesis of porous LaCoO_3_ nanospheres as supercapacitor electrode. Int J Electrochem Sci.

[33] Cheng X, Fabbri E, Nachtegaal M, Castelli IE, Kazzi ME, Haumont R, Marzari N, Schmidt TJ (2015). The oxygen evolution reaction on La1-xSrxCoO3 perovskites: A combined experimental and theoretical study of their structural, electronic, and electrochemical properties. Chem Mater.

[34] Liu K, Li J, Wang QF, Wang XP, Qian D, Jiang JB, Li JH, Chen ZH (2017). Designed synthesis of LaCoO_3_/N-doped reduced graphene oxide nanohybrid as an efficient bifunctional electrocatalyst for ORR and OER in alkaline medium. J Alloy Compd.

[35] Xu YJ, Wang LC, Cao PQ, Cai CL, Fu YB, Ma XH (2016). Mesoporous composite nickel cobalt oxide/graphene oxide synthesized via a template-assistant co-precipitation route as electrode material for supercapacitors. J Power Sources.

[36] Guan C, Liu JP, Cheng CW, Li HX, Li XL, Zhou WW, Zhang H, Fan HJ (2011). Hybrid structure of cobalt monoxide nanowire @ nickel hydroxidenitrate nanoflake aligned on nickel foam for high-rate supercapacitor. Energy Environ Sci.

[37] Wang XW, Zhu QQ, Wang XE, Zhang HC, Zhang JJ, Wang LF (2016). Structural and electrochemical properties of La_0.85_Sr_0.15_MnO_3_ powder as an electrode material for supercapacitor. J Alloy Compd.

[38] Tong H, Yue SH, Lu L, Jin FQ, Han QW, Zhang XG, Liu J (2017). A binder-free NiCo_2_O_4_ nanosheet/3D elastic N-doped hollow carbon nanotube sponge electrode with high volumetric and gravimetric capacitances for asymmetric supercapacitors. Nanoscale.

[39] Lü JB, Zhang YH, Lü Z, Huang XQ, Wang ZH, Zhu XB, Wei B (2015). A preliminary study of the pseudo-capacitance features of strontium doped lanthanum manganite. RSC Adv.

[40] Liu L, Zhang HJ, Fang L, Mu YP, Wang Y (2016). Facile preparation of novel dandelion-like Fe-doped NiCo_2_O_4_ microspheres@nanomeshes for excellent capacitive property in asymmetric supercapacitors. J Power Sources.

[41] Tomar AK, Singh G, Sharma RK (2018). Fabrication of a Mo-Doped Strontium Cobaltite Perovskite Hybrid Supercapacitor Cell with High Energy Density and Excellent Cycling Life. ChemSusChem.

[42] Yan D, Wang W, Luo X, Chen C, Zeng Y, Zhu ZH (2018). NiCo_2_O_4_ with oxygen vacancies as better performance electrode material for supercapacitor. Chem Eng J.

[43] Zhu YL, Zhou W, Sunarso J, Zhong YJ, Shao ZP (2016). Phosphorus-doped perovskite oxide as highly efficient water oxidation electrocatalyst in alkaline solution. Adv Funct Mater.

[44] Luan F, Wang GM, Ling YC, Lu XH, Wang HY, Tong YX, Liu XX, Li Y (2013). High energy density asymmetric supercapacitors with a nickel oxide nanoflake cathode and a 3D reduced graphene oxide anode. Nanoscale.

[45] Mo HY, Nan HS, Lang XQ, Liu SJ, Qiao L, Hu XY, Tian HW (2018). Influence of calcium doping on performance of LaMnO_3_ supercapacitors. Ceram Int.

[46] Alam M, Karmakar K, Pal M, Mandal K (2016). Electrochemical supercapacitor based on double perovskite Y_2_NiMnO_6_ nanowires. RSC Adv.

[47] Lang XQ, Mo HY, Hu XY, Tian HW (2017). Supercapacitor performance of perovskite La_1-x_Sr_x_MnO_3_. Dalton T.

[48] Cai WH, Lai T, Dai WL, Ye JS (2014). A facile approach to fabricate flexible all-solid-state supercapacitors based on MnFe_2_O_4_/graphene hybrids. J Power Sources.

[49] Li L, Bi HT, Gai SL, He F, Gao P, Dai YL, Zhang XT, Yang D, Zhang ML, Yang PP (2017). Uniformly dispersed ZnFe_2_O_4_ nanoparticles on nitrogen-modified graphene for high-performance supercapacitor as electrode. Sci Rep-UK.

[50] Wang ZF, Liu YS, Gao CW, Jiang H, Zhang JM (2015). A porous Co(OH)_2_ material derived from a MOF template and its superior energy storage performance for supercapacitors. J Mater Chem A.

[51] Liu PP, Liu J, Cheng S, Cai WZ, Yu FY, Zhang YP, Wu P, Liu ML (2017). A high-performance electrode for supercapacitors: silver nanoparticles grown on a porous perovskite-type material La_0.7_Sr_0.3_CoO_3-δ_ substrate. Chem Eng J.

[52] Huang L, Li Q, Zhang GG, Zhou X, Shao ZS, Zhou W, Cao JQ (2017). The preparation of LaSr_3_Fe_3_O_10-δ_ and its electrochemical performance. J Solid State Electr.

[53] Nagamuthu S, Vijayakumar S, Ryu KS (2017). Cerium oxide mixed LaMnO_3_ nanoparticles as the negative electrode for aqueous asymmetric supercapacitor devices. Mater Chem Phys.

